# 

**Published:** 1847-11

**Authors:** 


### CONTENTS

OF NO. V.

### ORIGINAL COMMUNICATIONS.

### ESSAYS AND CASES.

ART. I.—Notes on Medical Matters and Medical Men in Paris. By DAVID W. YANDELL, M.D., of Louisville, Ky. ........369

ART. II.—Typhus or Ship-Fever at Bellevue Hospital, New York. By B. F. WENDEL, M.D., one of the Assistant Physicians....396

ART. III.—Medical Reform. Reflections growing out of the Action of the National Convention of 1847. By W. L. SUTTON, M.D., of Georgetown, Kentucky........................403

ART. IV.—A Case of Tetanus—its Treatment and Cure. By T. B. GREENLEY, M.D., of West Point, Ky...................418

ART. V.—The Inhalation of Sulphuric Ether in Intermittent Fever and Tic Douloureux. By G. M. WHARTON, M.D., of Nashville, Tennessee.......................................423

### BIBLIOGRAPHICAL NOTICES.

ART. VI.—Observations on the Poisonous Properties of Sulphate of Quinine. By WILLIAM O. BALDWIN, M.D., of Montgomery, Alabama...............................................426

ART. VII.—A System of Surgery. By J. M. CHELIUS, Doctor in Medicine and Surgery, Public Professor of General and Ophthalmic Surgery, Director of the Chirurgical and Ophthalmic Clinic in the University of Heidelberg, etc. etc. Translated from the German, and accompanied with additional Notes and Observations. By JOHN F. SOUTH, late Professor of Surgery to the Royal College of Surgeons of England, and one of the Surgeons to St. Thomas's Hospital. Philadelphia: Lea and Blanchard. 3 vols. 8vo. 1847........436

ART. VIII.—The Institutes of Medicine. By MARTYN PAINE, A.M., M.D., Professor of the Institutes of Medicine and Materia Medica in the University of New York; Member of the Royal Verein fur Heilkunde in Preussen; of the Medical Society of Leipsic; of the Montreal Natural History Society, and other Learned Associations. NewYork: Harper and Brothers. 8vo. pp. 826. 1847 ....................440

ART. IX.—Lectures on the Principles and Practice of Physic, delivered at King's College, London. By THOMAS WATSON, M.D., Fellow of the Royal College of Physicians; late Physician to the Middlesex Hospital, and formerly Fellow of St. John's College, Cambridge. Third American, from the last London edition. Revised, with Additions, by D. FRANCIS CONDIE, M.D., Secretary of the College of Physicians; Author of a Treatise on the Diseases of Children, etc. Philadelphia: Lea and Blanchard. 1040 pp. 8vo. 1847...441

ART. X.—Outlines of the Veins and Lymphatics, with Short Descriptions. Designed for the Use of Medical Students. By JOHN NEIL, M.D., Demonstrator of Anatomy in the University of Pennsylvania; Lecturer on Anatomy in the Medical Institute of Philadelphia, etc. etc. Philadelphia: Ed. Barrington and G. D. Haswell. 29 pp. 8vo. 1847............442

ART. XI.—The American Medical Almanac for 1846; containing Statistics of the various Medical Colleges, Hospitals, Dispensaries, etc. of the United States, together with other information of value to the Physician and Student. Philadelphia: Lindsay and Blakiston..............................442

SELECTIONS FROM AMERICAN AND FOREIGN JOURNALS.

Prevention of Miscarriage and Premature Labor..................443
Union by the First Intention..............................443
Obituary Notice of Dr. Andrew Combe......................449
Neuralgia—Tic Douloureux..................................454

ORIGINAL INTELLIGENCE.

Traveling Letter from the Senior Editor.....................455
Medical Appointments......................................461
Ohio Medical Convention...................................462

